# Isolation and genome sequencing of five lytic bacteriophages from hospital wastewater in the Philippines

**DOI:** 10.1128/MRA.00311-23

**Published:** 2023-08-01

**Authors:** Michael Angelou L. Nada, Joseph B. Ancla, Nikka Mae R. Yadao, Virgilio P. de Paz, Jessica G. Manalaysay, Fred Lawrence D. Samante, Ursela G. Bigol

**Affiliations:** 1 Department of Science and Technology, Industrial Technology Development Institute, Taguig City, Philippines; Loyola University Chicago, Chicago, Illinois, USA

**Keywords:** whole-genome sequencing, bacteriophages, wastewater, philippines, phage therapy

## Abstract

Here, we report the genome sequences of five bacteriophages isolated from hospital wastewater, including two new species and two candidates for therapeutic application. No virulence, temperate marker and antibiotic resistance genes were found in the genomes of *Escherichia* phage vB_VIPECOOM03 and *Klebsiella* phage vB_VIPKPNUMC01, making them suitable candidate for therapy.

## ANNOUNCEMENT

Antimicrobial resistance is a global health concern. In Southeast Asia, infections with multidrug resistant (MDR) bacteria range from 4% to 68% (1), with bacteriophages recently explored to treat infection. Understanding phage diversity and genomic features is essential to develop an effective phage “cocktail” for therapeutic application. Here, we present the genome sequences of five bacteriophages isolated from hospital wastewater.

Sewage samples (1L) collected from Ospital ng Maynila Medical Center and De La Salle University Medical Center wastewater facilities were filtered using a 0.22-µm syringe filter and co-cultured (20 mL wastewater + 20 mL Luria-Bertani broth) with respective bacterial host (Table 1) for 24 h (agitation: 160 rpm). Phage isolation, purification, propagation, and titer determination were performed using plaque assay (2) and stored in SM buffer for further characterization.

**TABLE 1:** Genome characteristics and accession numbers of the five bacteriophage genomes^*a*^

Phage name	*Escherichia* phage vB_VIPECOOM03	*Enterobacter* phage vB_VIPECLOM01	*Enterobacter* phage vB_VIPECLUMC02	*Klebsiella* phage vB_VIPKPNUMC01	*Pseudomonas* phage vB_VIPPAEUMC01
Propagation host	*Escherichia coli* ATCC 25922	*Enterobacter cloacae* ATCC 13047	*Enterobacter cloacae* ATCC 13047	*Klebsiella pneumoniae* ATCC 13883	*Pseudomonas aeruginosa* ATCC 15442
No. of raw reads	3,132,575	2,984,666	2,141,041	2,004,323	8,875,898
No. of clean reads	2,702,757	2,798,181	1,891,580	1,812,001	8,031,297
Genome size (bp)	168,519	171,903	172,129	167,797	92,158
GC content (%)	35.49	39.83	39.80	39.55	49.35
Mean coverage	6,309	5,448	4,003	4,137	9,978
No. of CDS	269	288	287	279	173
No. of genes	279	306	305	295	188
No. of genes with predicted function	149 (53%)	148 (48%)	148 (48%)	147 (50%)	66 (35%)
No. of tRNAs	10	18	18	16	14
Head-neck tail organization	Myoviridae of neck type 2	Myoviridae of neck type 2	Myoviridae of neck type 2	Myoviridae of neck type 2	Myoviridae of neck type 1 (cluster 7)
Lifestyle prediction	Lytic	Lytic	Lytic	Lytic	Lytic
Temperate marker genes	–	Integrase (44,848–45,846)	Integrase (44,978–45,976)	–	cro gene (44,740–44,973)
Presence of antibiotic resistance genes	–	–	–	–	–
Presence of virulence genes	–	–	–	–	–
BioProject accession no.	PRJNA943845	PRJNA943928	PRJNA943930	PRJNA943931	PRJNA943933
SRA accession no.	SRR23866065	SRR23866658	SRR23866659	SRR23865899	SRR23865898
GenBank accession no.	OQ721911	OQ721912	OQ721913	OQ721914	OQ721915

^*a*^ “–” indicates the absence of genes in phage genomes.

Genomic DNA was extracted using phage DNA isolation kit (Norgen Biotek Corp., Thorold, ON, Canada). DNA libraries were prepared using Illumina DNA prep kit, and whole-genome sequencing was performed at DOST-ITDI virology laboratory using Illumina Miseq platform (2 × 250 bp PE), generating ~3,827,700 reads per sample. Read quality was assessed using FastQC v0.11.9 (https://www.bioinformatics.babraham.ac.uk/projects/fastqc/), trimmed with Trimmomatic v0.39 (3), and *de novo* assembled using Spades v3.14 (parameters: --careful --only-assembler -k 21,33,55,77,99,127) (4) followed by genome reorientation to reflect the rIIA/terL genes of the closest relative. To calculate genome coverage, reads were mapped back to the assembly using Bowtie2 v2.5.0 (5), indexed, and sorted with SAMtools v1.16.1 (6). Finally, genomes were polished using Pilon v1.24 (7), while quality and completeness were assessed using Quast v5.0.2 (8) and checkV v1.0.1 (9). Gene calling and annotations were conducted using Prokka v1.14.6 (10) utilizing PHROGs (11) database. Putative tRNAs and virion structural proteins were predicted using ARAGORN v1.2.41 (12) and STEP3 (13). Default parameters were used for all software unless otherwise specified.

Genome size ranges from 92,158 to 172,129 bp with GC content of 35.49%–49.35% and mean coverage of 4,003× to 9,978× (Table 1). CheckV (9) identified the genomes to be “complete” with direct terminal repeats and of high quality based on MIUViG criteria (14). *In silico* (15, 16) analysis predicted a lytic lifestyle and myoviridae-like morphology on all isolates; however, integrase and cro (repressor) genes were present in *Enterobacter* and *Pseudomonas* phage genomes suggesting access to temperate lifestyle. No virulence, toxin, or antibiotic resistance genes were detected using PhageLeads (17).

**Fig 1:**
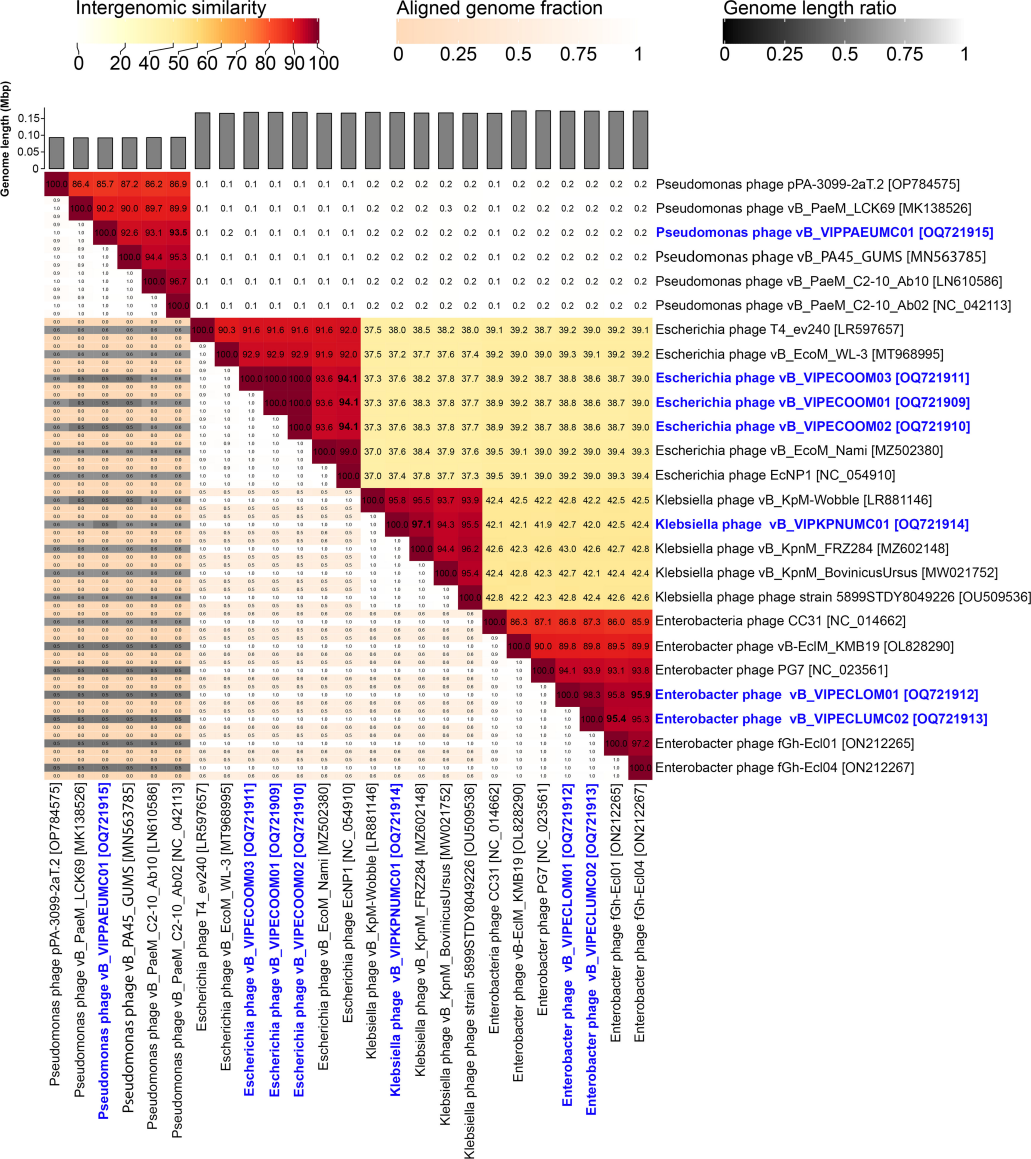
Comparative genome analysis of the assembled and reference genomes using Virus Intergenomic Distance Calculator. The five closest relative was determined using BLASTn search in NCBI. Darker color indicates high intergenomic similarities between genomes, with percent similarities (%) of the closest relative highlighted in bold. Genome sequence similarity of ≥95% is the same species. The number at the lower left part is alignment indicators. A high fraction (orange to white) of the genome is aligned in closely related phages and is expected to have similar genome length (black to white). The three coliphages (vB_VIPECOOM01, vB_VIPECOOM02, and vB_VIPECOOM03) isolated from the same location are identical species, and only *Escherichia* phage vB_VIPECOOM03 is reported here. The phage isolates and their accession numbers are in blue font.

For taxonomic classification, closely related genomes were obtained from NCBI database, and intergenomic similarities were computed using VIRIDIC v1.1 (Fig. 1) (18). Following the International Committee on Taxonomy of Viruses guidelines for demarcation of virus taxonomic ranks (19, 20), we identified *Escherichia* and *Pseudomonas* phages as novel species belonging to genus *Tequatrovirus* (94.1%) and *Pakpunavirus* (93.5%) and will be classified as “*Tequatrovirus vipecoom”* and “*Pakpunavirus vippaeumc*.” The *Enterobacter* and *Klebsiella* phages shared >95% genomic similarity to known phage genomes (Fig. 1).

Thus far, we identified two candidate phages for phage therapy which may further be explored to develop endolysin-derived antimicrobial agents. More research on phage biology and genomic characterization is still needed to develop a broad host-range therapeutic cocktail against MDR pathogens.

## Data Availability

The raw sequences (SRA) and genome assemblies were deposited in DDBJ/ENA/GenBank under the accession numbers listed in Table 1.
